# Supplementary material for: Transplant Candidates of 70+ Years Have Superior Survival If Receiving Pre-Emptively a Living Donor Kidney
Source: J Clin Med. 2024 Mar 23;13(7):1853. doi: 10.3390/jcm13071853 (PMC11012907; doi:10.3390/jcm13071853)
Supplement: Supplementary file 1 [file jcm-13-01853-s001.zip › jcm-2901913-supplementary.pdf]

Erasmus MC

Supplemental Material table of content:

Supplemental Table S1

Supplemental Table S2

Supplemental Table S1

Cause of death during follow-up of 349 recipients aged 70 years and older	
Cardiovascular disease	29 (20.7%)
malignancy	25 (17.8%)
infection	38 (27.1%)
other	10 (7.1%)
unknown	38 (27.1%)
Total number deceased recipients	140

Supplemental Table S2

Rejectionby Banff classification in kidney biopsies during follow-up of 349 recipients aged 70 years and older	
Borderline T cell mediated rejection	9 (11.6%)
T cell mediated rejection 1A	9 (11.6%)
T cell mediated rejection 1B	5 (6.5%)
T cell mediated rejection 2A	28 (36.4%)
T cell mediated rejection 2B	7 (9.1%)
T cell mediated rejection 3	3 (3.9%)
Active antibody-mediated rejection	13 (16.7%)
Chronic-active antibody-mediated rejection	3 (3.9%)
Total number of rejections	77
